# Atrial Fibrillation Burden Detected by Dual-Chamber Pacemakers as a Predictor for Cardiac Outcomes: A Retrospective Single-Center Cohort Study

**DOI:** 10.3389/fcvm.2021.654532

**Published:** 2021-06-25

**Authors:** Song-Yun Chu, Jie Jiang, Yu-Ling Wang, Qin-Hui Sheng, Jing Zhou, Yan-Sheng Ding

**Affiliations:** Department of Cardiology, Peking University First Hospital, Beijing, China

**Keywords:** atrial fibrillation burden, cardiac outcomes, pacemaker, readmission, heart failure

## Abstract

**Background:** Atrial fibrillation (AF) might lead to adverse cardiac consequences. The association between AF burden and cardiac prognosis is unknown.

**Methods and Results:** This retrospective cohort study enrolled 204 patients (117 males; age 74.5 ± 11.5 years) who underwent dual-chamber pacemaker implantation in our center from October 2003 to May 2017. During a median follow-up of 66.5 months, AF could be detected in 153 (75%) of the 204 pacemaker patients. Primary endpoint events (composite cardiac readmission, stroke or systemic embolism, and all-cause death) occurred in 83 cases (40.7%). In logistic regression analysis, AF detection was associated with increased risks of composite endpoints [odds ratio (OR) = 2.9, 95% confidence interval (CI): 1.3–6.2, *p* = 0.007], and the hazard was mainly driven by increased cardiac readmission (OR = 2.2, 95% CI: 1.1–4.7, *p* = 0.034). No significantly elevated risk for new-onset stroke, systemic embolism, or deaths were found in patients with AF detected than those without AF recorded. AF duration grade of more than 6 min suggested progressively increased composite endpoints (OR = 1.8, 95% CI: 1.2–2.7, *p* for trend = 0.005), cardiac readmission (OR = 1.8, 95% CI: 1.2–2.7, *p* for trend = 0.005), especially heart failure or acute coronary syndrome-associated readmission (OR = 1.8, 95% CI: 1.2–2.9, *p* for trend = 0.010), than those with shorter (<6 min) or no AF episodes. Kaplan–Meier analyses and Cox regression also suggested that episodes of AF more than 6 min predicted future cardiac events.

**Conclusions:** AF detected by pacemakers were common. Higher AF burden predicted more adverse cardiac outcomes and might suggest the intervention of rhythm control in these population.

## Introduction

Atrial fibrillation (AF) is the most common sustained arrhythmia, affecting 1–2% of the general population (1). Long-standing AF might promote the atrial remodeling process and leads to enlarged atria, cardiac dysfunction, and thromboembolic events. However, about 10–40% of all AF patients are asymptomatic, or so-called silent AF (2), and might be neglected for a long time until devastating cardiac outcomes developed. Additionally, paroxysmal AF is suggested not as only one entity. Outcomes may differ among different patterns and burden of AF; those with AF progression developed more cardiovascular events and all-cause mortality (3). Meanwhile, the lack of apparent symptoms and continuous monitoring makes it difficult to assess the AF burden in a clinical scenario. The threshold of AF burden to initiate anticoagulation and/or rhythm control also needs to be elucidated.

Implantable devices provide great opportunities to collect the data of AF burden in these patients. With data from published studies, the recent consensus document had addressed the clinical importance of device-detected atrial tachyarrhythmias (4, 5). To be noted, most prior studies only included events longer than 6 min, while the device-detected atrial tachyarrhythmia could be very short episodes. On the other hand, studies conducted using Holter data suggested that even short episodes of silent atrial tachycardia (AT) or AF conveyed an increased risk of stroke (4). There are still controversies regarding the optimal strategies for evaluating AF burden as well as initializing treatment (6, 7). We hypothesized that any AF detected by dual-chamber pacemakers might be used to predict adverse cardiac outcomes. We also attempted to explore the potential threshold of AF burden which might warrant intervention.

## Materials and Methods

### Study Population and Design

Consecutive adult (≥18 years) patients who underwent dual-chamber pacemaker implantation from October 2003 to May 2017 in our center were screened. To reduce the selection bias originated from different algorithms from different manufacturers, we only included patients with currently available models from Biotronik, Berlin, Germany (CYLOS DR, PROTOS DR CLS, Philos DR, Philos II DR, Evia DR, Estella DR, and Lumax 340 DR). Patients were routinely programmed at the first interrogation after implantation. Subjects who have pacemakers with a mode switch, atrial tachycardia detection, and intracardiac electrogram storage function activated, and the AF suppression functions deactivated throughout the follow-up period were included. If there was evidence of inappropriate AF detection or a pacemaker reprogramming that might impact AF detection, the subject was excluded from the analysis. Patients who were taking anticoagulants for any reason were excluded. The study was approved by the institutional review board of our hospital. Demographic, medical history, and echocardiographic data of the patients were captured from the electronic medical records. The medical history and outcomes were defined by the presence of diagnosis codes or prescription fills.

### Exposures—Atrial Fibrillation Detection and Burden Documented by Pacemaker

Pacemaker-detected AF was defined as a combined mode-switch (MSW) activation and/or high atrial rate (HAR) episode-based algorithm (8). For MSW episodes, the intervention rate was 160 bpm, and both onset and resolution criteria were five out of eight for all the models. AF episodes were also identified by HAR event-based algorithm. Specifically, for models Protos, Philos, Cylos, and Philos II, AF defined by HAR was a heart rate of more than 250 bpm, and the criteria for sudden-onset were met, but the criteria for stability were not. For models Evia, Estella, and Lumax, AF defined by HAR was a heart rate of more than 200 bpm, and onset and resolution criterion of 36 out of 48 and 20 out of 24 were met, respectively. EGM storage was further ascertained by blinded cardiologists to confirm the diagnosis and exclude artifacts or oversensing events according to the current consensus (4).

AF duration grade was defined as Grade 0, no AF episode was detected; Grade 1, AF episode(s) ≤ 6 min; Grade 2, AF episode(s) >6 min but ≤ 1 h; Grade 3, AF episode(s) >1 h but ≤ 24 h; Grade 4: AF episode(s) >24 h were detected during the follow-up, respectively. The longest AF episode was used to determine the AF duration grade if different duration of AF episodes were identified in one patient's record.

### Follow-Up and Outcomes

Patients were regularly followed-up with the interrogation record of the pacemaker and the document of the index outcomes. Device interrogations were conducted within 1 week, at 3 months, 6 months, and annually after implantation. The primary outcome was composite cardiac readmission, ischemic stroke or systemic embolism, and all-cause death, identified with evidence of a primary diagnosis during a hospitalization stay or an emergency department visit. Specific endpoint event was identified as: (1) cardiac readmission: subjects hospitalized for >24 h and discharged with the primary reason for admission listed as cardiovascular events in nature [i.e., arrhythmia, acute coronary syndrome (ACS), and heart failure]; (2) ischemic stroke or systemic embolism: supported by the consistency between symptoms and findings on brain/peripheral magnetic resonance imaging or computed tomography. For each outcome, only the first event of that outcome in a specific subject was included. For the composite outcome, only the first event in a given patient was included. To investigate the natural history of pacemaker-detected AF, participants were censored at the first of the following events: end of the follow-up or other end of study event (e.g., device explantation, death, or occurring of the endpoint events).

### Statistical Analysis

Continuous variables are expressed as mean ± SD or median and range for normal and skewed distributions. The Kolmogorov–Smirnov test was used to evaluate the normal distribution of continuous data. The *t*-test or the Mann–Whitney *U*-test was used to compare continuous variables between groups. Categorical variables were described by numbers or percentages. The chi-square test was used to test for categorical variables. The association between clinical characteristics, AF detected, and cardiac outcomes were assessed by the chi-square test. Univariate analysis was performed to preliminarily screen potential risk factors, including age, sex, past medical history (hypertension, diabetes mellitus, coronary heart disease, heart failure, stroke or systemic embolism, and peripheral arterial disease), echo-cardiac parameters, the indication of pacemaker implantation, and medications for index cardiac endpoints. Multivariate logistic regression models were then established to adjust the confounders of cardiac outcomes.

Survival was estimated by the Kaplan–Meier method, and differences in survival were evaluated with a log-rank test. Multivariate analyses with the Cox proportional-hazards model were used to assess the association of AF burden with cumulative risk for cardiac outcomes, with the results expressed as hazard ratios (HRs) with 95% confidence intervals (CIs).

Statistical analysis was performed using SPSS Statistics software, version 24.0 (IBM, Armonk, NY). Two-sided *p*-values < 0.05 were considered statistically significant.

## Results

### Patient Characteristics

We retrospectively evaluated 241 patients who were implanted with Biotronik pacemakers in our center between October 2003 and May 2017. Of these, we excluded 29 patients taking oral anticoagulants for any reason. Eight patients could not be contacted, so that no follow-up data could be collected. The final analysis included 204 pacemaker recipients (117 males; age 74.5 ± 11.5 years). Hypertension was the most prevalent comorbidities (73.0%), whereas diabetes (33.8%), coronary heart disease (33.8%), stroke (26.5%), and peripheral artery disease (16.2%) were less frequent. Heart failure history had rarely been documented (0.5%). The average atrial diameter [(3.8 ± 0.7) cm] and left ventricular ejection fraction [(66.5 ± 13.8)%] were within normal ranges. Medications included antiplatelet agents, beta-blockers, angiotensin-converting enzyme inhibitor (ACEI), and/or angiotensin receptor blockers (ARB), statins. Sick sinus syndrome was the primary indication for pacemaker implantation, accounting for 68.5% of the subjects (Table 1).

**Table 1:** Baseline characteristics of the patients.

**Characteristic**	**Overall (*N* = 204)**
**Demographic and history**	
Gender (male), n (%)	117 (57.4%)
Age (years) (mean ± SD)	74.5 ± 11.5
Diabetes, n (%)	69 (33.8%)
Hypertension, n (%)	149 (73.0%)
Coronary heart disease, n (%)	69 (33.8%)
Congestive heart failure, n (%)	1 (0.5%)
Peripheral artery disease, n (%)	33 (16.2%)
Stroke or systemic embolism history, n (%)	54 (26.5%)
CHADS_2_ score^a^ [n, interquartile range (IQR)]	2.0 (1.0–3.0)
CHA_2_DS_2_-Vasc score^b^ (n, IQR)	4.0 (3.0–5.0)
HASBLED score^c^ (n, IQR)	2.0 (1.0–3.0)
CHADS_2_ score ≥2 (n, %)	140 (68.6%)
CHA_2_DS_2_-Vasc score ≥2 (n, %)	187 (91.7%)
HASBLED score ≥3 (n, %)	63 (30.9%)
**Indication for pacemaker**	
Sick sinus syndrome (SSS), n (%)	134 (65.6%)
Atrial ventricular block (AVB), n (%)	56 (27.5%)
SSS+AVB, n (%)	6 (2.9%)
Others, n (%)	8 (3.9%)
**Echocardiography**	
Left atrial diameter^d^ (cm) [mean ± SD]	3.8 ± 0.7
LVEF (%) (mean ± SD)	65.6 ± 13.8
E/E′ (median, IQR)	11.8 (8.8–15.0)
**Medications**	
Anti-platelet (n, %)	98 (48.0%)
RAS inhibitor (n, %)	85 (41.7%)
Beta blocker (n, %)	81 (39.7%)
Statins (n, %)	92 (45.1%)

*SD, standard deviation; LVEF, left ventricular ejection fraction; E, peak early diastolic velocity of the mitral inflow; E', peak early diastolic velocity of the septal mitral annulus in tissue Doppler; RAS inhibitor, renin–angiotensin system inhibitor*.

a *CHADS_2_: Range from 0 to 6; higher score indicates higher risk of stroke. History of heart failure, hypertension, 75 years or older, and diabetes each is calculated as 1 point; prior stroke, TIA, or thromboembolism each is calculated as 2 points*.

b *CHA_2_DS_2_-Vasc: Range from 0 to 9; higher score indicates higher risk of stroke. History of heart failure, hypertension, diabetes, vascular disease, age 65–74 years, and female sex each is calculated as 1 point; 75 years or older and prior stroke, TIA, or thromboembolism each is calculated as 2 points*.

c *HASBLED: Range from 0 to 9; higher score indicates higher risk of bleeding. Point score is calculated as 1 point each for hypertension, abnormal kidney function, abnormal liver function, prior stroke, prior bleeding or bleeding predisposition, labile international normalized ratio (INR), older than 65 years, medication usage predisposing to bleeding, and alcohol use. In this study, our study population excluded those received anticoagulation therapy, thus the HASBLED score did not consider INR and the range is from 0 to 8*.

d *Left atrial diameter: left atrial anterior-posterior diameter measured using two-dimensional (2D) assessment in the parasternal long axis view*.

### Atrial Fibrillation Detected by Pacemaker and Atrial Fibrillation Burden Assessment

During a median follow-up of 66.5 [interquartile range (IQR) 45.0–90.0] months, AF episodes could be detected in 153 (75.0%) patients. Notably, in patients without clinical AF documented, 105 (69.1%) cases had AF episodes detected, and the distribution of AF episodes between patients with and without clinical AF history was diverse. Those with clinical AF history had more AF episodes lasting more than 1 h detected, while subjects without clinical AF history had most of their AF episodes lasting <6 min detected (Table 2).

**Table 2:** Incidence of pacemaker-detected atrial fibrillation (AF) and stratified by clinical AF history.

**Pacemaker-detected AF** ** (N, %)**	**Total** ** (*n* = 204)**	**Patients with AF history** **(*n* = 52)**	**Patients without AF history** ** (*n* = 152)**	***P*-value**
Any AF episodes	153 (75.0%)	48 (92.3%)	105 (69.1%)	<0.001^*^
AF lasting ≤ 6 min	72 (35.3%)	6 (11.5%)	66 (43.4%)	<0.001^*^
AF lasting >6 min, ≤ 1 h	22 (10.8%)	3 (5.8%)	19 (12.5%)	<0.001^*^
AF lasting >1 h, ≤ 24 h	32 (15.7%)	20 (38.5%)	12 (7.9%)	<0.001^*^
AF lasting >24 h	27 (13.2%)	19 (36.5%)	8 (5.3%)	<0.001^*^

* *P < 0.05*.

### Cardiac Outcomes and Atrial Fibrillation Burden Detected by Pacemakers

**Figure 1:**
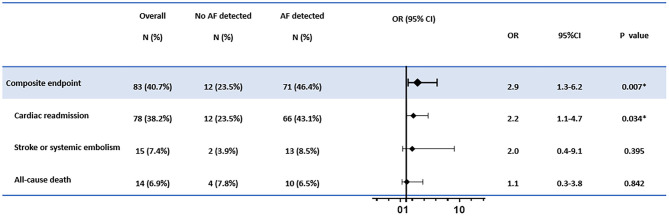
The association between atrial fibrillation (AF) detection and the cardiac outcomes. The adjusted confounders in the multivariate regression analysis for composite endpoint and cardiac readmission were age, sex, hypertension, indication for pacemaker implantation, and antiplatelet therapy; for stroke or systemic embolism were CHA_2_DS_2_-Vasc scores; for all-cause death were age, sex, and atrial fibrillation history, respectively. OR, odds ratio; CI, confidence interval. *p < 0.05.

The primary endpoint of the composite cardiac outcomes occurred in 83 cases (40.7% of the cohort). For individual events, cardiac readmission was observed in 78 cases (38.2%), stroke or systemic embolism was observed in 15 cases (7.4%), and 14 cases (6.9%) died (Figure 1).

In multivariate logistic regression, AF detection was associated with increased risks of composite endpoints (OR = 2.9, 95% CI: 1.3–6.2, *p* = 0.007). The elevated hazard was mainly driven by increased cardiac readmission (OR = 2.2, 95% CI: 1.1–4.7, *p* = 0.034). In contrast, no significantly elevated risks for new-onset stroke, systemic embolism, or deaths were found in patients with AF detected than those without AF recorded (Figure 1).

**Figure 2:**
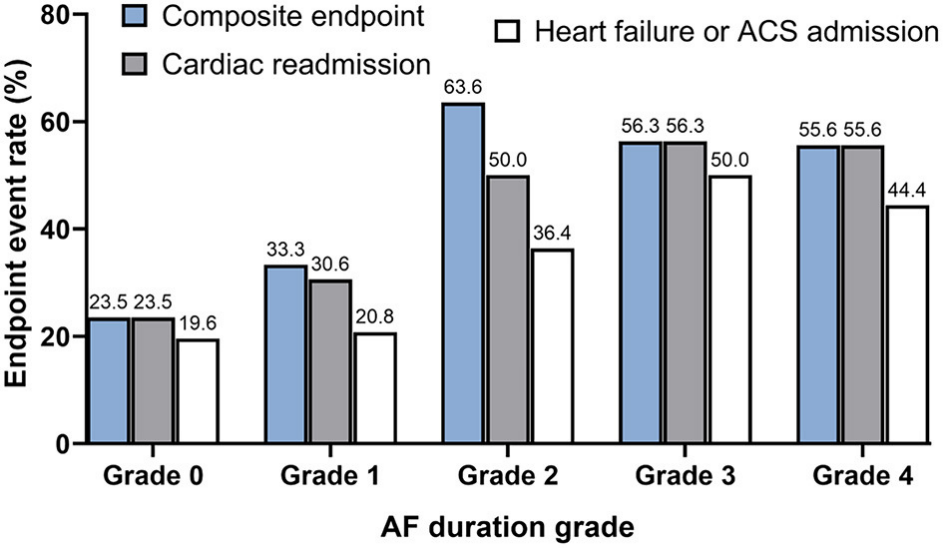
The distribution of composite endpoint, cardiac readmission, and heart failure or acute coronary syndrome-associated admission stratified by pacemaker-detected AF duration grade. Note the trend of progressively increased cardiac outcomes with the AF grade escalated from Grades 0 to 2, although the occurrence of the events kept at a steady state after the duration of AF detected longer than 6 min were detected (Grades 2, 3, and 4). AF duration grade was defined as Grade 0: No AF episode was detected; Grade 1: only AF episode(s) ≤ 6 min was detected; Grade 2: AF episode(s) >6 min but ≤ 1 h was detected; Grade 3: AF episode(s) >1 h but ≤ 24 h detected; Grade 4: AF episode(s) >24 h was detected.

Further analysis of the relationship between the endpoint events and AF burden was conducted. While there was only a minor increase in the primary composite events and cardiac readmission when short-episode ( ≤ 6 min) AFs (AF duration grade 1) were detected, significantly increased endpoint events when AF was longer than 6 min (AF duration grades 2–4) were recorded. However, no significant difference in the occurrence of endpoint events were found between subjects with AF duration of grades 2, 3, and 4. Also, among all the reasons for re-admission, heart failure or acute coronary syndrome other than arrhythmia events increased with the AF grade escalated by a similar trend (Figure 2).

**Figure 3:**
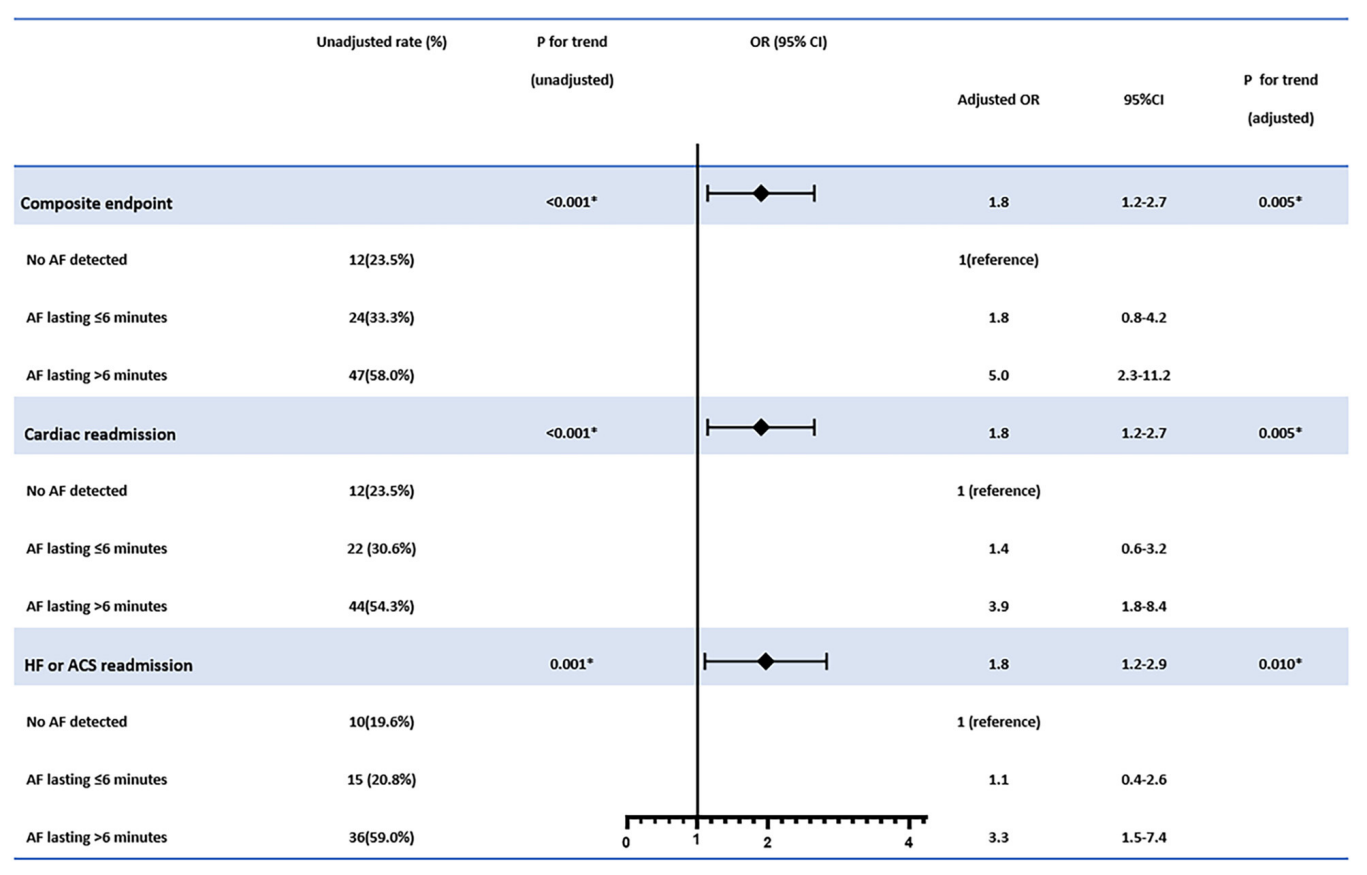
The association between atrial fibrillation (AF) burden detected and the cardiac outcomes. The adjusted confounders in the multivariate regression analysis for composite endpoint were age, sex, stroke or systemic embolism history, and antiplatelet therapy; for cardiac readmission were age, sex, clinical AF history, stroke or systemic embolism history, and antiplatelet therapy; for heart failure or ACS admission were age, sex, hypertension, coronary heart disease, AF history, stroke or systemic embolism history, and antiplatelet therapy, respectively. OR, odds ratio; CI, confidence interval. *p < 0.05.

In multivariate logistic regression, AF duration of 6 min or more showed stepwise elevated risks for composite endpoints (OR = 1.8, 95% CI: 1.2–2.7, *p* for trend = 0.005), cardiac readmission (OR = 1.8, 95% CI: 1.2–2.7, *p* for trend = 0.005), and heart failure or ACS-associated admission (OR = 1.8, 95% CI: 1.2–2.9, *p* for trend = 0.010) than those with short episodes ( ≤ 6 min) of AF and no AF at all (Figure 3).

**Figure 4:**
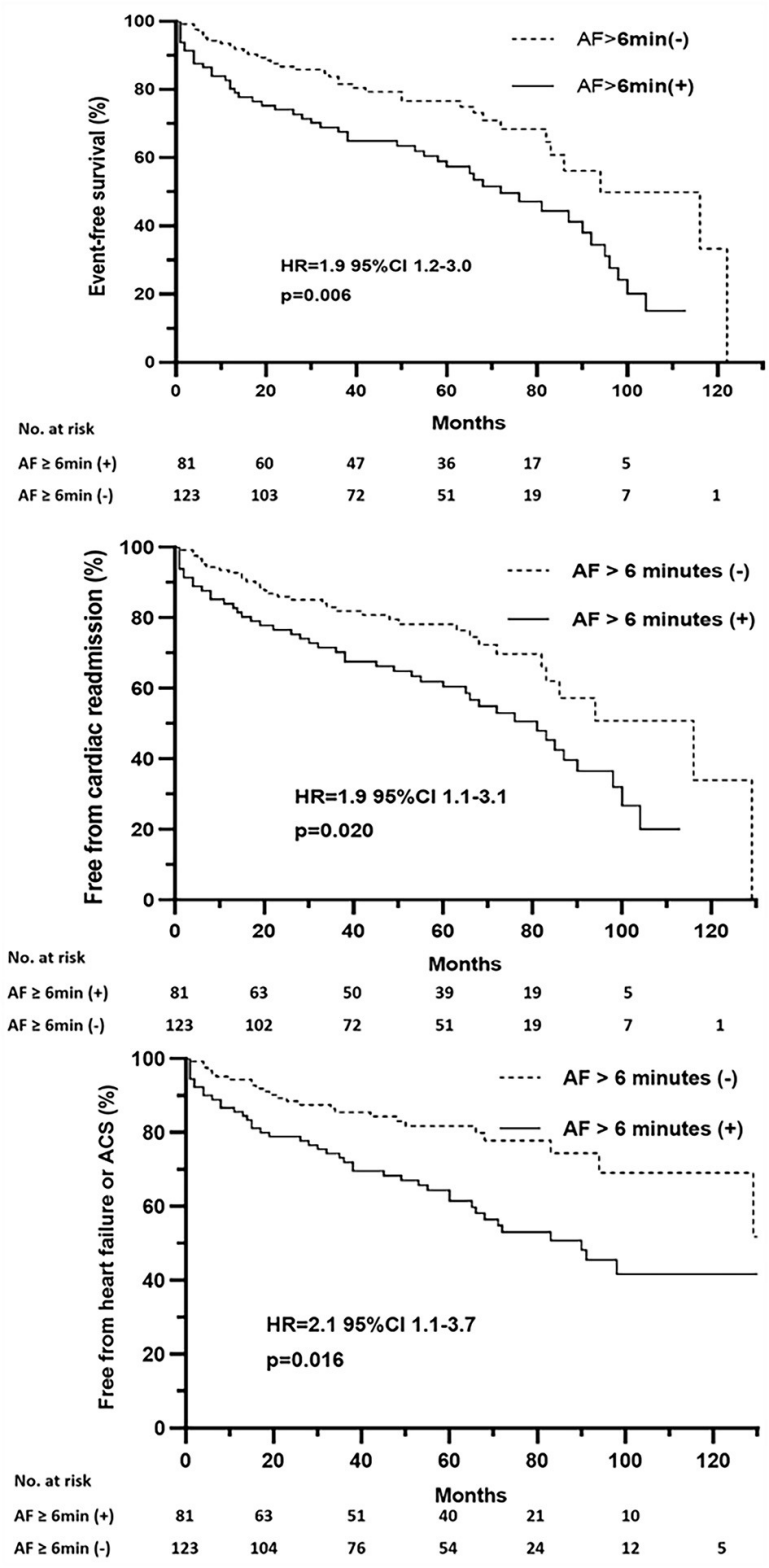
Event-free survival after pacemaker implantation stratified by pacemaker-detected AF episodes of more than 6 min. HR, hazard ratio; CI, confidence interval.

The survival analysis showed a significant correlation between a more than 6-min episode of AF detection and a shorter event-free survival time. Multivariate Cox proportional hazards analysis further showed that episodes of AF more than 6 min were associated with increased risk of composite endpoints (HR = 1.9, 95% CI 1.2–3.0, *p* = 0.006), cardiac readmission (HR = 1.9, 95% CI 1.1–3.1, *p* = 0.020), and heart failure or ACS-associated admission (HR = 2.1, 95% CI 1.1–3.7, *p* = 0.016) (Figure 4).

## Discussion

### Main Findings

The detection of AF episodes was common in patients who underwent dual-chamber pacemaker implantation. Most of the AF episodes detected by pacemakers were short runs. Subjects with any AF detected had increased risks for adverse cardiac outcomes. Moreover, the impact of AF on cardiac events increased with the AF burden escalated to more than 6 min.

### Detection of Atrial Fibrillation Episodes by Implantable Devices

The effort to assess the AF burden in real-world clinical practice has often been hampered by the variation of paroxysmal attacks and the poor correlation between symptoms (if any) and true episodes of arrhythmias. Routine 12-lead ECG as a screening tool usually yields little. Longer time monitoring by Holter or intermittent handheld ECG recordings identifies AF better (9). Cardiac implantable electronic devices (CIEDs) have the advantage of longer monitoring time and screening algorithms with intracardiac electrograms to identify high atrial rate (HAR) events.

Earlier studies (10–12) defined the device-detected AF as HAR events sustained for a certain duration (usually more than 5 or 6 min) in fear of the false positives from the far-field R wave sensing and noises (13). These measures, however, might also lead to leaving out real AF events with shorter duration or slower ventricular rate. In the present study, we adopted the combined definition of MSW and HAR algorithm as well as EGM evidence aiming to collect more accurate AF burden, as we have demonstrated the feasibility and accuracy (8).

AF episodes could be detected in our cohort in as many as three quarters. Even in patients without clinical AF history, the AF detection rate was up to ~70%. The higher detection rate might result from the inclusion of any duration of AF episodes and a more extended follow-up period. The so-called “subclinical” AF incidence was ~30% if we only included episodes >6 min in patients without AF history as early studies, which was consistent with prior reports (12, 14). More AF episodes recorded in patients without clinical AF history though were short runs ( ≤ 6 min). In contrast, the AF episodes shown in patients with clinical AF history were more prolonged (>1 h). The diverse distribution of the AF episodes might reflect the different total AF burden in patients with and without clinical AF history.

### Cardiac Outcomes and Atrial Fibrillation Detected by Pacemakers

Any AF detection was associated with a 2-fold increased risk of composite endpoints, and the elevated hazard was mainly driven by increased cardiac readmission. The analysis between AF duration grade and cardiac outcomes also suggested a progressively elevated risk for the composite endpoint, cardiac readmission, especially heart failure or ACS-associated admission when AF episode sustained for more than 6 min. AF episodes lasting more than 6 min also predicted a shorter event-free survival. We believe that any AF contributes to the adverse outcomes, although the longer duration of AF weighs more. No significantly elevated risks for new-onset stroke, systemic embolism, or deaths were found in patients with AF detected than those without AF recorded.

The critical cutoff for AF duration associating with stroke hazard is still unclear. A prior study conducted using Holter data suggested that even short episodes of silent AF convey an increased risk of stroke (15). On the other hand, short episodes (<15–20 s) of AF were not associated with increased risk of clinical events in the RATE Registry (16). The subclinical AF burden for predicting stroke risk varied among studies, from 5 to 6 min [MOST (17) and ASSERT (14)], 1 h, >5.5 h daily [SOS AF (18) and TRENDS (19)] to >24 h [AT500 Registry (20)]. In a meta-analysis of >10,000 subjects with CIEDs, the risk of stroke only increased in patients with a minimum episode of AF >5 min (18). In our study, most of the AF episodes were short runs. The lack of correlation between AF detection and the embolism events might partially result from the scarcity of longer episodes detected. The influence of the short-run AF needs to be clarified in more large-scale studies. Besides, the patients in our cohort had ~70% with a CHADS_2_ score ≥2 and more than 90% with a CHA_2_DS_2_-Vasc score ≥2, which suggested clinical characteristics indicating high stroke or systemic embolic risks. In this scenario, the AF burden might not act as a potent risk factor sufficient to act as an add-on effect (8). The result might be an extrapolation of earlier studies such as SPORTIF III and V, which reported similar stroke rates in patients with paroxysmal and persistent AF and at least two risk factors for stroke (21).

The close relationship between AF detection and heart failure or ACS-associated admission is impressive. Rarely clinical heart failure history had been recorded, and an average of normal ejection fraction was noted at baseline in this population. Considering the advanced age and high prevalence of hypertension in the cohorts, these might reflect the impact of the rapid and irregular rhythm of AF on diastolic functional reserve. Besides, sick sinus syndrome (SSS) as the indication for pacemaker implantation were more found in our patients with AF detected than those without AF just as seen in earlier reports (10, 22–24), which might indicate that the degenerative atrial remodeling or fibrotic atrial cardiomyopathy serves as the common substrate of impaired heart function and AF development (25). As for ACS, most of the patients underwent coronary angiography, yet no obstructive coronary artery was found. The finding was consistent with the prior study, which suggested the more prevalent AF for myocardial infarction with no obstructive coronary artery (MINOCA) (26). The ischemic chest discomfort symptoms and/or elevated cardiac biomarker might suggest slow coronary blood flow or micro-emboli as the underlying pathophysiological mechanism.

### Perspective: Possible Intervention for Atrial Fibrillation Detected by Pacemakers

As mentioned above, the critical cutoff of AF burden associated with increased risk of stroke varied among different studies. There are still controversies for initializing anticoagulation for subclinical AF (27, 28). In the present study, most of the AF episodes detected were short runs, and no significant elevated risks for stroke or systemic embolism was found. In fact, about 90% of the patients had CHA_2_DS_2_-Vasc score ≥2, and no one received anticoagulants while the incidence of new-onset stroke was rather low. Our result was in line with earlier data (29), which suggested that patients with device-detected AF seem to be at lower thrombotic risk than the general AF population. As stated in the latest ESC guidelines, the absolute risk of stroke associated with subclinical AF may be lower than with clinical AF (5, 30, 31). The AF burden could be a marker of the severity of atrial cardiomyopathy and stroke risk instead of acting as a cause (4, 28, 32). Meanwhile, anticoagulation based on subclinical AF recurrences did not improve the outcome (33). More evidence is needed in future large-scale clinical trials. Continued follow-up and monitoring to detect progression to clinical AF, or subclinical AF burden transition to longer durations, as well as underlying comorbidity change are warranted (5, 34).

In contrast, AF detection by the pacemaker associated with the elevated risk for heart failure or ACS, and AF lasting more than 6 min, seemed to be enough to contribute to the outcomes. These might suggest that these patients may benefit more from rhythm control. Recently, the generalizability of the CASTLE-AF trial indicated that ablation therapy might benefit patients regardless of the ejection fraction (35). Upstream therapy, such as angiotensin receptor blockers, β-blockers, and statins, might also be considered.

### Limitation

This study is a single-center retrospective cohort study, and the sample size is small. Pacemaker default settings for AF detection were slightly different between different models, although devices of only one manufacturer were included. Therefore, the results may not directly extrapolate to other practice settings. The scarcity of endpoint events led to less power in the statistical calculation and difficult for further sub-analysis.

## Conclusion

Detection of AF episodes was common in patients with dual-chamber pacemakers implanted. The composite cardiac adverse outcomes, cardiac re-admission, especially for heart failure, or ACS, were associated with pacemaker-detected AF. Episodes of AF lasting more than 6 min showed not only further elevated risks for the endpoint events than those with short-run AF and no AF at all, but also suggested future cumulative hazard.

## Data Availability Statement

All data needed to evaluate the conclusions in the paper are present in the paper.

## Ethics Statement

This retrospective cohort study was approved by the Ethics Committee on Clinical Investigation of Peking University First Hospital. Considering the retrospective nature of the present study, a waiver of informed consent from patients was obtained. All the procedures being performed were part of the routine care.

## Author Contributions

S-YC, JZ, and JJ formed the conception and designed the study. S-YC, Y-LW, Q-HS, and JZ analyzed and interpreted the data. S-YC, Q-HS, JJ, and JZ drafted the manuscript. JJ, Q-HS, JZ, Y-LW, S-YC, and Y-SD revisited it critically for important intellectual content. All the authors have read and given the final approval of the manuscript submitted.

## Conflict of Interest

The authors declare that the research was conducted in the absence of any commercial or financial relationships that could be construed as a potential conflict of interest.
